# Factor Structure and Psychometric Properties of the Body Perception Questionnaire–Short Form (BPQ-SF) Among Chinese College Students

**DOI:** 10.3389/fpsyg.2020.01355

**Published:** 2020-06-30

**Authors:** Nantong Wang, Fen Ren, Xiaolu Zhou

**Affiliations:** ^1^Research Institute for International and Comparative Education, Shanghai Normal University, Shanghai, China; ^2^School of Education and Psychology, University of Jinan, Jinan, China

**Keywords:** body perception, body awareness, autonomic reactivity, Chinese college students, factor structure, psychological properties

## Abstract

**Objective:**

Body perception, including body awareness and reactivity, is featured in a range of mental health conditions. However, research on Chinese questionnaires assessing body perception has been surprisingly absent. The present study aimed to investigate the factor structure and psychometric properties of the Body Perception Questionnaire–Short Form (BPQ-SF) among Chinese.

**Methods:**

the current sample included 688 Chinese college students. Self-report scales were used to measure body perception, somatization, and depressive somatic and psychological symptoms.

**Results:**

Confirmatory factor analysis supported a three-factor model of the BPQ-SF, involving body awareness, supradiaphragmatic reactivity, and subdiaphragmatic reactivity. Good internal consistency and test–retest reliability were observed. Convergent validity was established by significant correlations with scores of somatization and somatic symptoms of depression. Divergent validity was evidenced by non-significant association with ratings on psychological symptoms of depression. The very short form of the body awareness subscale of BPQ can be an alternative to the body awareness subscale when scale length is the priority.

**Conclusion:**

The BPQ-SF possessed three latent factors and demonstrated good psychometric properties that can measure body perception among Chinese in a reliable and valid way.

## Introduction

Body perception refers to the perception of physical experiences from inside the body. It arises from a complex network of afferent and efferent neural pathways between organs/tissues and the central nervous system (Porges, 1993; Cameron, 2001; Craig, 2002). Body perception includes a variety of physical experiences such as pain, dyspnea, stomach discomfort, and muscle tension. Disrupted body perception is featured in several mental health conditions (Khalsa et al., 2018), notably depression (Harshaw, 2015), anxiety disorders (Paulus and Stein, 2010), and somatic symptom disorders (Dimsdale et al., 2013). Some conceptual models hold that these disorders are fundamentally disorders of body perception (Paulus and Stein, 2010; Khalsa and Lapidus, 2016). Moreover, cultural studies suggest that heightened body perception is related to higher degree of somatic symptom emphasis, that is, somatization, among non-Westerners, including Chinese (Ryder et al., 2008), Korean (Zhou et al., 2015), African (Dzokoto and Adams, 2005), and Cambodian (Hinton et al., 2007), in the presence of psychological distress (Ma-Kellams, 2014). Examining the relation between body perception and these psychopathological concerns requires proper assessment of body perception. However, few measurements of body perception have been subjected to rigorous psychometric testing (Mehling et al., 2009).

### Measures of Body Perception

Multiple methods have been employed to assess body perception: self-report body or interoceptive awareness scales (Mehling et al., 2009) and psychophysiological measures such as heartbeat detection tasks (Barrett et al., 2004; Wiens, 2005; Fairclough and Goodwin, 2007), as well as neuroimaging measures including functional magnetic resonance imaging (Schulz, 2016). Comparing with the objective psychophysiological and neuroimaging measures, self-report questionnaires are easily affected by recall bias or poor insight. However, self-report scales still have the unique advantages in both clinical settings and laboratories: they are convenient to use, allowing capturing information from multiple organs and, more importantly, helping conceptually connecting subjective body perception to physiological and neural processes (Cabrera et al., 2018). Surprisingly, in Chinese culture where body perception comprises a central part of several mental health concerns, research on Chinese questionnaires of body perception has so far been absent. A good start point is to translate a well-established scale into Chinese and test its factor structure and psychometric properties among Chinese populations.

Mehling et al. (2009) reviewed extant self-report measures of body perception and found that different scales targeted different aspects of body perception. Some attend to normal, non-emotive bodily process (e.g., Body Awareness Questionnaire, Shields et al., 1989); some focus on the physical sensation characteristic of certain mental disorder (e.g., Body Vigilance Scale, Schmidt et al., 1997), and some emphasize the attitude to body sensations (e.g., Body Responsiveness Questionnaire, Daubenmier, 2005). For the purpose of exploring the relation between body perception and broadband mental health problems, we choose to translate and test the Body Perception Questionnaire–Short Form (BPQ-SF, Cabrera et al., 2018), which assesses perceived (negative) bodily experiences and responses in relation to autonomic nervous system, in a Chinese college student sample.

### Body Perception Questionnaire–Short Form

The BPQ-SF keeps two (body awareness and autonomic nervous system reactivity) of five subscales from the original BPQ (Porges, 1993). Reasons of this adaptation are as follows: (1) the original BPQ is lengthy (122 items), limiting its application; (2) the retained body awareness and autonomic nervous system reactivity subscales have been found to be most interested by other researchers; some studies even use only these two subscales (Critchley et al., 2004; Bernatova and Svetlak, 2017); (3) the three deleted subscales target stress coping and health history, which have been assessed by other widely used questionnaires. Not all items of the original body awareness and autonomic nervous system reactivity subscales are retained. Item selection is based on theoretical consideration and results of exploratory factor analysis (Cabrera et al., 2018). The final version of the body awareness subscale consisted of 26 items, and the final version of the autonomic nervous system reactivity subscale comprises 20 items.

Using online and college student data from Spain and the United States, Cabrera et al. (2018) identified a three-factor structure from the two subscales: items of the body awareness subscale loaded on one factor, named body awareness (BA), whereas items of the autonomic nervous system reactivity subscale clustered into two factors, named supradiaphragmatic reactivity (SUPR) and subdiaphragmatic reactivity (SUBR). The three factors are conceptualized by the organization of the autonomic neural pathways. Body awareness describes the sensitivity of bodily signals, emerging from the converged afferent pathways. Supradiaphragmatic reactivity and SUBR reflect the unique effects of ventral vagal complex (VVC) and dorsal vagal complex (DVC). According to the polyvagal theory (Porges, 1995, 2009, 2011), VVC and DVC are two distinct circuits within the parasympathetic nervous system. The VVC comprises efferent pathways originating in the nucleus ambiguus, regulating striated muscles of the face and head, as well as organs above the diaphragm, whereas the DVC contains efferent pathways originating in the dorsal motor nucleus, regulating visceral organs below the diaphragm.

In addition to the three-factor structure, Cabrera et al. (2018) also found that the BPQ-SF showed good reliability (categorical omega coefficient and test–retest reliability) and converged with measures of stress and somatosensory amplification, in both Spanish and American samples. This evidence preliminarily suggests the cross-culture property of the scale. Furthermore, considering the research situations in which the scale length is the priority, Cabrera et al. (2018) developed a very short form of BA subscale, named the Body Perception Questionnaire–Body Awareness Very Short Form (BPQ-VSF), by *post hoc* analyses. Specifically, the authors tested if 10 to 15 items with highest factor loadings across the Spanish and American data sets can yield scores having adequate overlap with the 26-item score. The authors used ρ = 0.90 as criterion and found that the lowest item count was twelve items. The BPQ-VSF showed acceptable internal consistency (categorical omega coefficient, Cabrera et al., 2018).

### The Present Study

The current study examines the factor structure and psychometric properties of the BPQ-SF among Chinese college students. We hypothesize that (1) the three correlated BA, SUPR, and SUBR factor structure of the BPQ-SF will be validated in our Chinese sample; (2) the BPQ-SF will demonstrate good internal consistency and test–retest reliability; (3) the BPQ-SF will be significantly associated with scores of somatization and somatic/bodily symptoms of depression (convergent validity), but not with psychological symptoms of depression (divergent validity); (4) the BPQ-VSF will obtain good internal consistency, test–retest reliability, and convergent and divergent validity in our Chinese sample. The BPQ-VSF score will have adequate overlap with the 26-item BA subscale score.

## Materials and Methods

### Participants

Participants were 688 participants from different classes (e.g., psychology, electronic information engineering, and primary school education) and different grades at a public university in China. We chose to recruit college students for two reasons: students were evidenced as heterogeneous as the community people (Hanel and Vione, 2016) and the facility of collecting test–retest data.

### Procedures

The study was approved by the Shanghai Normal University Research Ethics Committee. Research assistants administered the survey at the end of the courses. Participants provided written informed consent and then completed a paper-and-pencil battery of questionnaires, which took approximately 30 min. All participants received a notebook as a gift for reimbursing their time.

### Measures

#### BPQ-SF (Cabrera et al., 2018)

The BPQ-SF is answered on a five-point Likert scale ranging from 1 = *never* to 5 = *always*. A total score is calculated across all items. The original BPQ-SF is in English. Iterations of translation and back-translation of the measure were conducted by independent bilingual psychological major postgraduates until satisfactory Chinese version was achieved. Psychological properties of this measure were detailed in the results session.

#### Self-Rating Depression Scale (SDS, Zung, 1965)

The SDS is a 20-item self-report measure assesses somatic (SOM) and psychological (PSY) symptoms of depression. Respondents answer on a 4-point scale ranging from 1 (*a little of time*) to 4 (*most of the time*). Separate subscale scores for somatic symptoms and psychological symptoms can be garnered (Passik et al., 2000). The SDS has demonstrated good psychometric properties among Chinese clinical patients, non-clinical community people, and college students (Liu and Dai, 1994; Duan and Sheng, 2012; Liu et al., 1995). In the current study, Cronbach’s α of SDS was 0.83.

#### Symptom Checklist 90 (SCL-90, Derogatis et al., 1973)

The SCL-90 is a 90-item measure that assesses self-report symptoms of psychological distress, including somatization. Respondents rate on a 5-point scale from 1 (*not at all*) to 5 (*severe*). The SCL-90 has demonstrated sound psychometric properties among Chinese community people (Chen and Li, 2003; Jin et al., 1986). The current study used only the somatization subscale. This subscale assesses the tendency to experience somatic symptoms in response to psychological distress (Carrozzino et al., 2017; Carrozzino et al., 2018; Carrozzino et al., 2016). In this study, Cronbach’s α of somatization subscale was 0.91.

### Data Analysis

To investigate the factor structure of the BPQ-SF, responses to the individual items were subjected to confirmatory factor analysis (CFA), using Mplus 7.0 (Muthén and Muthén, 2015) with WLSMV (weighted least squares means and variance-adjusted estimation). WLSMV was used because it is suitable for categorical items with five (as with the case of the BPQ-SF) or fewer options (Finney and DiStefano, 2006; Bandalos, 2014). A three-factor model, with correlated BA, SUPR, and SUBR factors, was contrasted with a two-factor model, with BA and a general autonomic reactivity factor. Three fit indices were adopted to assess the goodness-of-fit of the CFA models: the comparative fit index (CFI), the Tucker-Lewis index (TLI), and the root mean square error of approximation (RMSEA) with its 90% confidence intervals (CIs). Overall fit for the indices was evaluated using these criteria (Hu and Bentler, 1999): CFI and TLI acceptable if ≥0.90 and good if ≥0.95; RMSEA acceptable if ≤0.08 and good if ≤0.06.

Descriptive statistics, including means, standard deviations, and ranges, were calculated with SPSS 21.0 (IBM Corp, 2012). Internal consistencies were assessed by computing categorical omega coefficients (categorical ω, Green and Yang, 2008; Kelley and Pornprasertmanit, 2016), with the MBESS R package (Kelley, 2019); 95% CIs of categorical omega coefficients were calculated, using bias-corrected and accelerated bootstrapping with 1,000 draws. Categorical omega coefficient is suitable when factor loadings are variable, and the items are categorical, as with the case of the BPQ-SF (McNeish, 2018). Test–retest reliability was assessed by Pearson correlation.

Convergent validities were assessed by calculating the Pearson correlation with scores of somatization and partial correlation with SDS somatic symptoms subscale score, controlling the SDS psychological symptoms subscale score. Divergent validities were assessed by computing partial correlation with the score of SDS psychological symptoms subscale, controlling the SDS somatic symptoms score. Partial correlations were applied because of the conceptual and empirical (*r* = 0.42, *p* < 0.001) association between psychological and somatic symptoms of depression.

*Post hoc* analyses were conducted to test internal consistency, test–retest reliability, and convergent and divergent validity of the BPQ-VSF created by Cabrera et al. (2018). The descriptive statistics of the BPQ-VSF were reported. Beside, Pearson correlation between the BPQ-VSF score and the 26-item BA subscale score was computed to test the overlap between the two versions.

## Results

### Demographic Characteristics for the Study Sample

Participants included 688 college students. The mean age of the sample was 19.85 years (range, 17–29 years; SD, 1.87), and 53.3% (*n* = 367) were males. Approximately 44.8% of the participants were freshmen, 27.5% were sophomore, 14.8% were junior, 3.6% were senior, and 8.6% were postgraduate. The majority of participants were Han (96.4%); the other participants represented eight minorities, including Yi, Hui, Tujia, Miao, and Zang, among others.

### Factor Structure of the BPQ-SF

The three-factor CFA model showed acceptable fit: CFI = 0.90, TLI = 0.90, RMSEA = 0.06, 90% CI = [0.055–0.059]. As shown in Table 1, all observed variables were loaded on the three correlated latent variables. The item “I feel like vomiting” loaded on both SUPR and SUBR factors. The standardized correlations between the three factors were as follows: 0.77 for BA and SUPR, 0.71 for BA and SUBR, and 0.65 for SUPR and SUBR, *p*s < 0.001. The two-factor CFA model did not have good model fit: CFI = 0.88; TLI = 0.87; RMSEA = 0.06, 90% CI = [0.06–0.07]. Therefore, Cabrera et al. (2018) three-factor structure was supported in the present sample.

**TABLE 1:** Confirmatory factor analysis standardized factor loadings of BPQ-SF.

Items	Factor loading
BA01	0.48***
BA02	0.47***
BA03	0.57***
BA04	0.57***
BA05	0.60***
BA06	0.50***
BA07	0.60***
BA08	0.50***
BA09	0.59***
BA10	0.65***
BA11	0.59***
BA12	0.65***
BA13	0.66***
BA14	0.67***
BA15	0.48***
BA16	0.57***
BA17	0.61***
BA18	0.62***
BA19	0.54***
BA20	0.44***
BA21	0.65***
BA22	0.57***
BA23	0.50***
BA24	0.69***
BA25	0.65***
BA26	0.63***
SUPR01	0.65***
SUPR02	0.53***
SUPR03	0.67***
SUPR04	0.65***
SUPR05	0.77***
SUPR06	0.74***
SUPR07	0.67***
SUPR08	0.70***
SUPR09	0.66***
SUPR10	0.68***
SUPR11	0.69***
SUPR12	0.69***
SUPR13	0.66***
SUPR14	0.63***
SUPB	0.34***/0.38***
SUBR02	0.80***
SUBR03	0.78***
SUBR04	0.80***
SUBR05	0.82***
SUBR06	0.78***

BA, body awareness; SUPR, supradiaphragmatic reactivity; SUBR, subdiaphragmatic reactivity; SUPB, item “I feel like vomiting.” ***p < 0.001.

### Descriptive Statistics of the BPQ-SF

Descriptive statistics (i.e., mean, SD, and range) for the BPQ-SF and the three dimensions are shown in Table 2.

**TABLE 2:** Descriptive statistics of BPQ-SF, three subscales, and Body Awareness Very Short Form.

	Mean	SD	Range
BPQ-SF	83.21	19.04	24–144
BA	50.43	11.46	0–86
SUPR	23.87	7.03	0–50
SUBR	10.53	3.58	0–23
BPQ-VSF	22.35	5.50	11–42

*BPQ-SF, Body Perception Questionnaire–Short Form; BA, body awareness; SUPR, supradiaphragmatic reactivity; SUBR, subdiaphragmatic reactivity; BPQ-VSF, Body Perception Questionnaire–Body Awareness Very Short Form.*

### Internal Consistency of the BPQ-SF

The internal consistencies for the total score and three subscale scores were as follows: the total categorical ω = 0.94, 95% CI [0.93–0.94]; BA subscale categorical ω = 0.90, 95% CI [0.87–0.91]; SUPR subscale categorical ω = 0.88, 95% CI [0.86–0.89]; and SUBR subscale categorical ω = 0.85, 95% CI [0.82–0.87].

### Test–Retest Reliability of the BPQ-SF

Eighty-three participants (42.2% male) finished the BPQ-SF 1 month after the first test. Good test–retest reliabilities were observed for the BPQ-SF total score (*r* = 0.78, *p* < 0.01) and the three subscales scores (*r*_BA_ = 0.72, *r*_SUPR_ = 0.71, and *r*_SUBR_ = 0.74, *ps* < 0.01).

### Convergent and Divergent Validity of the BPQ-SF

Pearson correlations used to investigate convergent and divergent validity are shown in Table 3. In testing convergent validity, medium positive relationships were observed between the BPQ-SF and somatization, as well as SDS-SOM. The association between the BPQ-SF and SDS-SOM remains significant, after controlling the effects of SDS-PSY. In examining divergent validity, a weak relationship was observed between the BPQ-SF and SDS-PSY, and the relationship was no longer significant when the SDS-SOM score was controlled (*r* = 0.06, *p* = 0.14).

**TABLE 3:** Pearson correlations of convergent and divergent validity.

	Somatization	SDS-SOM		SDS-PSY	
		Bivariate correlations	Partial correlations	Bivariate correlations	Partial correlations
BPQ-SF	0.49**	0.32**	0.27**	0.19**	0.06
BA	0.42**	0.29**	0.24**	0.16**	0.05
SUPR	0.48**	0.27**	0.22**	0.19**	0.09
SUBR	0.37**	0.24**	0.21**	0.11**	0.01
BPQ-VSF	0.42**	0.28**	0.23**	0.18**	0.07

BPQ-SF, Body Perception Questionnaire–Short Form; BA, body awareness; SUPR, supradiaphragmatic reactivity; SUBR, subdiaphragmatic reactivity; BPQ-VSF, Body Perception Questionnaire–Body Awareness Very Short Form; Somatization, the subscale of SCL-90; SDS, Self-Rating Depression Scale; SDS-PSY/-SOM, psychological/somatic symptoms subscale of SDS. **p < 0.01.

### *Post hoc* Analysis: The BPQ-VSF

The standardized factor loadings of the 12 items of the BPQ-VSF were high in the present data set, ranging from 0.57 to 0.69. The Pearson correlation between this 12-item score and the 26-item score was 0.94, meeting the statistical criterion (ρ = 0.90). Descriptive statistics of the BPQ-VSF are shown in Table 2. Internal consistency was good, categorical ω = 0.84, 95% CI [0.82–0.85]. Test–retest reliability was acceptable, *r* = 0.68. When evaluating convergent validity, there were medium positive relation with somatization and significant association with SDS-SOM after controlling SDS-PSY score. In examining divergent validity, there was a weak association with SDS-PSY, and the correlation was no longer significant when SDS-SOM score was controlled (Table 3).

## Discussion

This study represents the first attempt to test a self-report questionnaire of body perception among Chinese people. The BPQ-SF demonstrated good psychometric properties in the present sample. It is also notable that the BPQ-VSF was found to be a good alternative to the BA subscale of the BPQ-SF among Chinese college students. This version provides convenience when scale length is of uppermost concern.

Consistent with the findings in Spanish and American people (Cabrera et al., 2018), the Chinese BPQ-SF comprised three correlated factors: BA, SUPR, and SUBR. We have already introduced the conceptualization of the separate three factors. Worthy of attention are the high correlations between the three factors. These high correlations, to some extent, are theoretically reasonable. The high correlations between the BA and the two autonomic nervous system reactivity factors (SUPR and SUBR) might be due to the feedback loops between the afferent and efferent pathways. The high relation between the SUPR and SUBR factors could reflect the effects of sympathetic system. Coordinating with the parasympathetic efferent system, the sympathetic efferent pathways innervate the same organs both above and below the diaphragm, thus contributing to the strength of the relation between SUPR and SUBR (Cabrera et al., 2018). Besides, the high correlations might be partly due to random sampling variability. In the study of Cabrera et al. (2018), the correlations fluctuated in different samples from the same culture background. Additional data are needed to further examine the relations between the three latent factors.

In the present study, the item “I feel like vomiting” was equally cross-loaded on the SUPR and SUBR factors. Compared with Cabrera et al. (2018), our result is consistent with the finding from the Spanish sample and inconsistent with the results from the American samples. The item was more related to SUBR versus SUPR in the American samples. Cabrera et al. (2018) attributed the differences to culture differences. Data from more culture groups are needed to test the hypothesis.

Convergent validity of the BPQ-SF was established by positive associations with ratings of somatization and somatic symptoms of depression. This could be due to the function of autonomic neural pathways, upon which the BPQ-SF is developed. It is through those pathways that all organs and tissues, including those inferred in items of the somatization and SDS somatic symptoms subscale, connect to higher brain structures, thus leading to related bodily awareness and responses. Divergent validity of the BPQ-SF was supported by non-significant correlation with the psychological symptoms of depression, when controlling for the effects of somatic depressive symptoms. The findings that psychological and somatic symptoms of depression were differently related to the BPQ-SF are in line with evidence from neuroscience. For example, Avery et al. (2014) found that the abnormal hemodynamic activities in the dorsal mid-insula (the primary cortex for various bodily “feelings”) were correlated with somatic depressive symptoms, rather than psychological complaints, in people with depression.

Our findings provided preliminary evidences for the validity and reliability of the BPQ-SF among Chinese, however, several limitations should be acknowledged. First, the college student sample limited the generalizability of the results. In order to apply the measure to clinical settings, future studies should recruit clinical samples. Second, the constructs used to test the convergent and divergent validities of the BPQ-SF were assessed by self-report measures. It is necessary to test the validity of the BPQ-SF against objective measures, such as heart rate detection accuracy or neural imaging activities. Third, the present study examined only the psychometric properties of the BPQ-SF and did not evaluate the clinimetric features of the scale. Future studies are needed to test the clinimetric properties, particularly the clinical validity, scalability, and sensitivity, of this scale (Carrozzino, 2019; Fava et al., 2018). Fourth, the present study validated the BPQ-VSF by *post hoc* analyses. Future studies should test this version through more strict methodological steps, as suggested by Smith et al. (2000).

The current study served as a small but important step in understanding body perception, as well as the relation between body perception and mental health concerns. Body perception has been linked with emotional experience, decision making, self-regulation, homeostatic control, and motivation (Khalsa et al., 2018). Body perception is regarded as crucial for understanding the psychopathology (Duquette, 2017). Some researchers proposed that dysfunctional body perception is causally linked to the symptomatology (Harshaw, 2015). Moreover, body perception has been recently discussed as a new biomarker in psychiatry (Khalsa and Lapidus, 2016). Exploring the different associations between the three factors of the BPQ-SF and various clinical variables could help in understanding the exact role body perception plays in a range of psychiatric disorders. In the recent years, development and testing of body perception treatments for different mental health problems have been frequently discussed (Khalsa et al., 2018). To apply the BPQ-SF in the clinical practice, more evidences for the psychometric properties, clinimetric properties, and clinical utilities of the BPQ-SF are needed. The current study contributed by providing Chinese college student data. Last but not least, the Chinese BPQ-SF can be used in the cross-culture studies between Chinese and people of west European heritage and help in exploring the cultural differences of body perception and clarifying the relation between body perception and somatization under different cultural contexts.

## Data Availability Statement

The datasets generated for this study are available on request to the corresponding author.

## Ethics Statement

The studies involving human participants were reviewed and approved by Shanghai Normal University Research Ethics Committee. The patients/participants provided their written informed consent to participate in this study.

## Author Contributions

NW collected the data, did data analysis, and drafted the manuscript. FR did data analysis and revised the manuscript. XZ contributed to the conception and design of the study, as well as revised the manuscript. All authors contributed to the article and approved the submitted version.

## Conflict of Interest

The authors declare that the research was conducted in the absence of any commercial or financial relationships that could be construed as a potential conflict of interest.
